# Crystal structure of 3-[4-(1*H*-imidazol-1-yl)phen­yl]-2-(4-nitro­phen­yl)prop-2-ene­nitrile

**DOI:** 10.1107/S2056989015013730

**Published:** 2015-08-06

**Authors:** Ting-ting Yu, Ming-Di Yang, Jing-jing Pi, Yu-Bin Zhang, Jian-Hua Yu

**Affiliations:** a Deparment of Chemistry, Anhui University, Hefei 230039, People’s Republic of China; b Key Laboratory of Functional Inorganic Materials, Chemistry, Hefei 230039, People’s Republic of China

**Keywords:** crystal structure, delocalised *D*—π—*A* electronic structure, hydrogen bonding

## Abstract

In the title compound, C_18_H_12_N_4_O_2_, which has a delocalized *D*—π—*A* electronic structure, the dihedral angles between the central benzene ring and the planes of the pendant imidazole and nitro­benzene rings are 37.65 (9) and 4.96 (7)°, respectively. In the centrosymmetric crystal structure, mol­ecules are linked by weak C—H⋯O inter­actions, generating [001] *C*(6) chains.

## Related literature   

For chemical and photophysical background, see: Liu *et al.* (2006 ▸); Zheng *et al.* (2013 ▸). For a related structure, see: Li (2011 ▸).
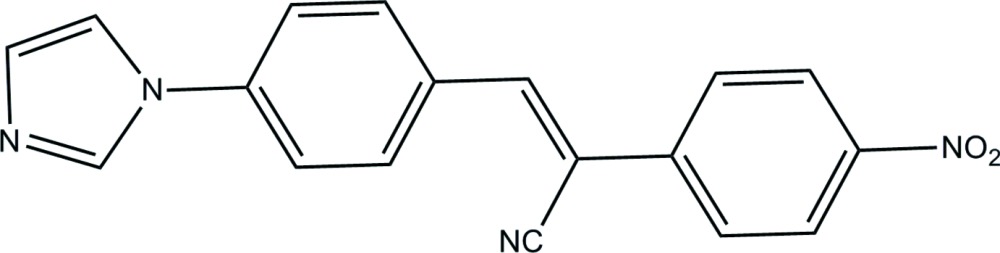


## Experimental   

### Crystal data  

C_18_H_12_N_4_O_2_
*M* *_r_* = 316.32
Monoclinic, 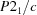

*a* = 7.1792 (16) Å
*b* = 16.512 (4) Å
*c* = 12.771 (3) Å
β = 101.557 (3)°
*V* = 1483.3 (6) Å^3^
*Z* = 4
Mo *K*α radiation
μ = 0.10 mm^−1^
*T* = 296 K
0.25 × 0.2 × 0.18 mm

### Data collection  

Bruker SMART CCD diffractometer
Absorption correction: multi-scan (*SADABS*; Bruker, 2000 ▸) *T*_min_ = 0.320, *T*_max_ = 0.439
10392 measured reflections
2609 independent reflections
2081 reflections with *I* > 2σ(*I*)
*R*_int_ = 0.024

### Refinement  

*R*[*F* ^2^ > 2σ(*F* ^2^)] = 0.036
*wR*(*F* ^2^) = 0.105
*S* = 1.07
2609 reflections
217 parameters
H-atom parameters constrained
Δρ_max_ = 0.18 e Å^−3^
Δρ_min_ = −0.17 e Å^−3^

### 

Data collection: *SMART* (Bruker, 2000 ▸); cell refinement: *SAINT* (Bruker, 2000 ▸); data reduction: *SAINT*; program(s) used to solve structure: *SHELXTL* (Sheldrick, 2008 ▸); program(s) used to refine structure: *SHELXTL*; molecular graphics: *SHELXTL*; software used to prepare material for publication: *SHELXTL*.

## Supplementary Material

Crystal structure: contains datablock(s) I, Global. DOI: 10.1107/S2056989015013730/hb7432sup1.cif

Structure factors: contains datablock(s) I. DOI: 10.1107/S2056989015013730/hb7432Isup2.hkl

Supporting information file. DOI: 10.1107/S2056989015013730/hb7432Isup3.cml

**Fig. 1.**
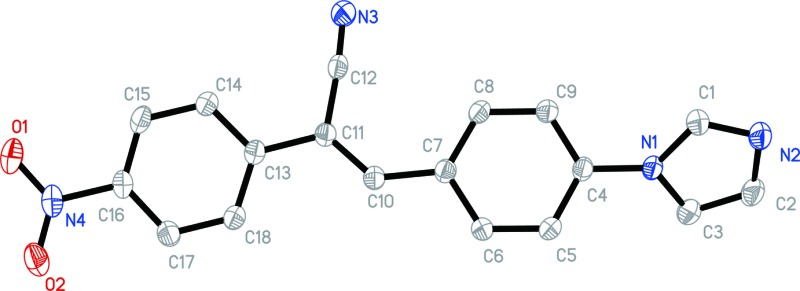
The molecular structure of the title molecule.

. DOI: 10.1107/S2056989015013730/hb7432fig1.tif
The mol­ecular structure of the title mol­ecule.

**Fig. 2.**
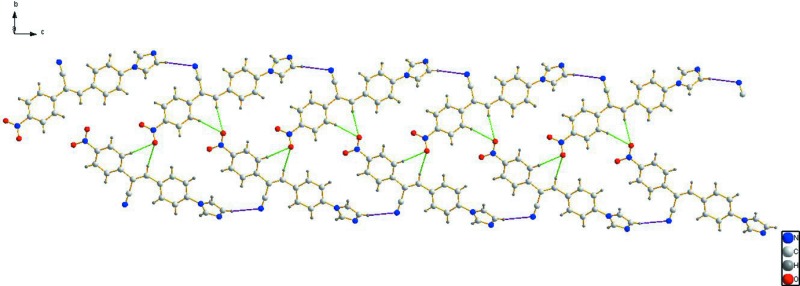
The extended structure of the title compound.

. DOI: 10.1107/S2056989015013730/hb7432fig2.tif
The extended structure of the title compound.

CCDC reference: 1045501

Additional supporting information:  crystallographic information; 3D view; checkCIF report

## Figures and Tables

**Table 1:** Hydrogen-bond geometry (, )

*D*H*A*	*D*H	H*A*	*D* *A*	*D*H*A*
C18H18O2^i^	0.93	2.54	3.464(2)	173

Symmetry code: (i) 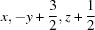.

## supplementary crystallographic information

### S1. Synthesis and crystallization

3-(4-Imidazol-1-yl-phenyl)-2-(4-nitro-phenyl)-acrylo­nitrile was dissolved in ethanol solvent. Then added 4-nitro-benzo­nitrile into the solvent. When the two compounds were mixed completly, dropwise added a few piperidine into them. About seven hours later, we could get the title compound.

### S2. Refinement

Crystal data, data collection and structure refinement details are summarized in Table 1. All hydrogen atoms were placed in geometrically idealized positions and constrained to ride on their parent atoms, with C—H = 0.93 Å and *U*_iso_(H) = 1.2 *U*_eq_.

### Crystal data

**Table:**

C_18_H_12_N_4_O_2_	*Z* = 4
*M**_r_* = 316.32	*F*(000) = 656
Monoclinic, *P*2_1_/*c*	*D*_x_ = 1.416 Mg m^−^^3^
Hall symbol: -P 2ybc	Mo *K*α radiation, λ = 0.71073 Å
*a* = 7.1792 (16) Å	µ = 0.10 mm^−^^1^
*b* = 16.512 (4) Å	*T* = 296 K
*c* = 12.771 (3) Å	Block, red
β = 101.557 (3)°	0.25 × 0.2 × 0.18 mm
*V* = 1483.3 (6) Å^3^	

### Data collection

**Table:**

Bruker SMART CCD diffractometer	2609 independent reflections
Radiation source: sealed tube	2081 reflections with *I* > 2σ(*I*)
Graphite monochromator	*R*_int_ = 0.024
ω scans	θ_max_ = 25.0°, θ_min_ = 2.0°
Absorption correction: multi-scan (*SADABS*; Bruker, 2000)	*h* = −8→8
*T*_min_ = 0.320, *T*_max_ = 0.439	*k* = −19→17
10392 measured reflections	*l* = −15→15

### Refinement

**Table:**

Refinement on *F*^2^	Primary atom site location: structure-invariant direct methods
Least-squares matrix: full	Secondary atom site location: difference Fourier map
*R*[*F*^2^ > 2σ(*F*^2^)] = 0.036	Hydrogen site location: inferred from neighbouring sites
*wR*(*F*^2^) = 0.105	H-atom parameters constrained
*S* = 1.07	*w* = 1/[σ^2^(*F*_o_^2^) + (0.0531*P*)^2^ + 0.2091*P*] where *P* = (*F*_o_^2^ + 2*F*_c_^2^)/3
2609 reflections	(Δ/σ)_max_ = 0.002
217 parameters	Δρ_max_ = 0.18 e Å^−^^3^
0 restraints	Δρ_min_ = −0.17 e Å^−^^3^

### Special details

**Table:**

Geometry. All e.s.d.'s (except the e.s.d. in the dihedral angle between two l.s. planes) are estimated using the full covariance matrix. The cell e.s.d.'s are taken into account individually in the estimation of e.s.d.'s in distances, angles and torsion angles; correlations between e.s.d.'s in cell parameters are only used when they are defined by crystal symmetry. An approximate (isotropic) treatment of cell e.s.d.'s is used for estimating e.s.d.'s involving l.s. planes.
Refinement. Refinement of *F*^2^ against ALL reflections. The weighted *R*-factor *wR* and goodness of fit *S* are based on *F*^2^, conventional *R*-factors *R* are based on *F*, with *F* set to zero for negative *F*^2^. The threshold expression of *F*^2^ > σ(*F*^2^) is used only for calculating *R*-factors(gt) *etc*. and is not relevant to the choice of reflections for refinement. *R*-factors based on *F*^2^ are statistically about twice as large as those based on *F*, and *R*- factors based on ALL data will be even larger.

### Fractional atomic coordinates and isotropic or equivalent isotropic displacement parameters (Å^2^)

**Table:**

	*x*	*y*	*z*	*U*_iso_*/*U*_eq_	
N1	0.33343 (18)	0.36839 (7)	0.53687 (9)	0.0409 (3)	
C11	0.2512 (2)	0.49937 (9)	0.03244 (11)	0.0373 (3)	
C16	0.1639 (2)	0.65848 (9)	−0.23484 (11)	0.0433 (4)	
C7	0.2711 (2)	0.47837 (9)	0.23353 (10)	0.0380 (3)	
C14	0.2517 (2)	0.52940 (9)	−0.15944 (11)	0.0441 (4)	
H14	0.2917	0.4765	−0.1669	0.053*	
C9	0.3390 (2)	0.36047 (9)	0.34742 (11)	0.0412 (4)	
H9	0.3709	0.3059	0.3557	0.049*	
C4	0.3103 (2)	0.40518 (9)	0.43461 (10)	0.0379 (3)	
C13	0.2195 (2)	0.55534 (8)	−0.06072 (11)	0.0370 (3)	
C5	0.2614 (2)	0.48643 (9)	0.42196 (11)	0.0458 (4)	
H5	0.2416	0.5167	0.4802	0.055*	
C10	0.2475 (2)	0.52285 (9)	0.13288 (11)	0.0404 (4)	
H10	0.2257	0.5780	0.1395	0.048*	
C6	0.2421 (2)	0.52205 (9)	0.32260 (11)	0.0438 (4)	
H6	0.2091	0.5765	0.3147	0.053*	
C8	0.3203 (2)	0.39658 (9)	0.24824 (11)	0.0421 (4)	
H8	0.3408	0.3661	0.1904	0.050*	
N4	0.1372 (2)	0.71310 (10)	−0.32681 (11)	0.0563 (4)	
C18	0.1554 (2)	0.63447 (9)	−0.05281 (11)	0.0441 (4)	
H18	0.1308	0.6527	0.0120	0.053*	
C15	0.2251 (2)	0.58088 (10)	−0.24634 (11)	0.0481 (4)	
H15	0.2484	0.5632	−0.3117	0.058*	
O2	0.0835 (2)	0.78255 (8)	−0.31500 (10)	0.0736 (4)	
O1	0.1702 (2)	0.68742 (9)	−0.41145 (10)	0.0818 (5)	
C17	0.1280 (2)	0.68607 (9)	−0.13928 (12)	0.0461 (4)	
H17	0.0859	0.7388	−0.1331	0.055*	
N3	0.3111 (3)	0.35246 (9)	−0.02129 (11)	0.0666 (5)	
C12	0.2850 (2)	0.41644 (10)	0.00623 (11)	0.0448 (4)	
N2	0.4589 (2)	0.29296 (8)	0.67745 (10)	0.0579 (4)	
C1	0.4684 (2)	0.31340 (9)	0.57988 (12)	0.0496 (4)	
H1	0.5579	0.2926	0.5436	0.059*	
C3	0.2306 (3)	0.38325 (10)	0.61467 (12)	0.0562 (5)	
H3	0.1281	0.4184	0.6103	0.067*	
C2	0.3083 (3)	0.33654 (10)	0.69857 (12)	0.0603 (5)	
H2	0.2653	0.3342	0.7625	0.072*	

### Atomic displacement parameters (Å^2^)

**Table:**

	*U*^11^	*U*^22^	*U*^33^	*U*^12^	*U*^13^	*U*^23^
N1	0.0543 (8)	0.0397 (7)	0.0291 (6)	−0.0003 (6)	0.0094 (5)	0.0024 (5)
C11	0.0401 (8)	0.0386 (8)	0.0334 (7)	−0.0035 (6)	0.0078 (6)	0.0014 (6)
C16	0.0444 (9)	0.0498 (9)	0.0355 (8)	−0.0052 (7)	0.0075 (6)	0.0098 (7)
C7	0.0425 (8)	0.0386 (8)	0.0322 (7)	−0.0036 (6)	0.0062 (6)	−0.0001 (6)
C14	0.0549 (10)	0.0432 (8)	0.0363 (8)	0.0002 (7)	0.0137 (7)	−0.0001 (6)
C9	0.0526 (9)	0.0356 (8)	0.0356 (8)	−0.0009 (7)	0.0097 (7)	0.0000 (6)
C4	0.0441 (9)	0.0398 (8)	0.0295 (7)	−0.0026 (6)	0.0066 (6)	0.0015 (6)
C13	0.0376 (8)	0.0414 (8)	0.0319 (7)	−0.0054 (6)	0.0071 (6)	0.0008 (6)
C5	0.0640 (11)	0.0425 (9)	0.0312 (7)	0.0033 (7)	0.0100 (7)	−0.0040 (6)
C10	0.0490 (9)	0.0375 (8)	0.0348 (8)	−0.0009 (6)	0.0084 (6)	0.0018 (6)
C6	0.0600 (10)	0.0355 (8)	0.0354 (8)	0.0036 (7)	0.0080 (7)	0.0014 (6)
C8	0.0556 (9)	0.0402 (8)	0.0315 (7)	−0.0008 (7)	0.0116 (6)	−0.0033 (6)
N4	0.0608 (9)	0.0647 (10)	0.0443 (8)	−0.0025 (8)	0.0127 (7)	0.0175 (7)
C18	0.0555 (10)	0.0446 (9)	0.0333 (7)	−0.0009 (7)	0.0115 (7)	−0.0002 (6)
C15	0.0576 (10)	0.0577 (10)	0.0315 (8)	−0.0041 (8)	0.0152 (7)	0.0016 (7)
O2	0.0926 (11)	0.0641 (9)	0.0665 (8)	0.0148 (7)	0.0215 (7)	0.0280 (7)
O1	0.1185 (12)	0.0900 (10)	0.0419 (7)	0.0087 (9)	0.0283 (7)	0.0193 (6)
C17	0.0548 (10)	0.0412 (8)	0.0420 (8)	−0.0010 (7)	0.0085 (7)	0.0040 (6)
N3	0.1093 (14)	0.0488 (9)	0.0411 (8)	0.0102 (8)	0.0134 (8)	−0.0023 (7)
C12	0.0594 (10)	0.0468 (10)	0.0280 (7)	−0.0003 (8)	0.0081 (7)	0.0032 (6)
N2	0.0896 (11)	0.0457 (8)	0.0374 (7)	0.0032 (7)	0.0100 (7)	0.0066 (6)
C1	0.0659 (11)	0.0437 (9)	0.0390 (8)	0.0049 (8)	0.0102 (7)	0.0048 (7)
C3	0.0763 (12)	0.0558 (10)	0.0417 (9)	0.0094 (9)	0.0247 (8)	0.0039 (7)
C2	0.0971 (14)	0.0522 (10)	0.0362 (8)	−0.0022 (10)	0.0245 (9)	0.0046 (7)

### Geometric parameters (Å, º)

**Table:**

N1—C1	1.361 (2)	C13—C18	1.395 (2)
N1—C3	1.3736 (19)	C5—C6	1.380 (2)
N1—C4	1.4197 (17)	C5—H5	0.9300
C11—C10	1.3455 (19)	C10—H10	0.9300
C11—C12	1.442 (2)	C6—H6	0.9300
C11—C13	1.4876 (19)	C8—H8	0.9300
C16—C15	1.372 (2)	N4—O1	1.2277 (18)
C16—C17	1.375 (2)	N4—O2	1.2287 (18)
C16—N4	1.4626 (19)	C18—C17	1.377 (2)
C7—C6	1.3971 (19)	C18—H18	0.9300
C7—C8	1.399 (2)	C15—H15	0.9300
C7—C10	1.4606 (18)	C17—H17	0.9300
C14—C15	1.381 (2)	N3—C12	1.141 (2)
C14—C13	1.3939 (19)	N2—C1	1.306 (2)
C14—H14	0.9300	N2—C2	1.370 (2)
C9—C8	1.3820 (19)	C1—H1	0.9300
C9—C4	1.3853 (19)	C3—C2	1.347 (2)
C9—H9	0.9300	C3—H3	0.9300
C4—C5	1.388 (2)	C2—H2	0.9300
			
C1—N1—C3	105.65 (12)	C5—C6—C7	121.66 (14)
C1—N1—C4	126.83 (13)	C5—C6—H6	119.2
C3—N1—C4	127.46 (13)	C7—C6—H6	119.2
C10—C11—C12	122.11 (13)	C9—C8—C7	121.00 (13)
C10—C11—C13	123.67 (13)	C9—C8—H8	119.5
C12—C11—C13	114.22 (12)	C7—C8—H8	119.5
C15—C16—C17	121.71 (13)	O1—N4—O2	123.44 (14)
C15—C16—N4	118.88 (13)	O1—N4—C16	118.48 (15)
C17—C16—N4	119.41 (15)	O2—N4—C16	118.08 (14)
C6—C7—C8	117.63 (12)	C17—C18—C13	121.15 (14)
C6—C7—C10	116.55 (13)	C17—C18—H18	119.4
C8—C7—C10	125.82 (12)	C13—C18—H18	119.4
C15—C14—C13	121.12 (14)	C16—C15—C14	118.97 (14)
C15—C14—H14	119.4	C16—C15—H15	120.5
C13—C14—H14	119.4	C14—C15—H15	120.5
C8—C9—C4	120.27 (13)	C16—C17—C18	119.00 (15)
C8—C9—H9	119.9	C16—C17—H17	120.5
C4—C9—H9	119.9	C18—C17—H17	120.5
C9—C4—C5	119.76 (13)	N3—C12—C11	175.48 (15)
C9—C4—N1	120.17 (13)	C1—N2—C2	104.24 (14)
C5—C4—N1	120.06 (12)	N2—C1—N1	112.88 (15)
C14—C13—C18	118.02 (13)	N2—C1—H1	123.6
C14—C13—C11	120.40 (13)	N1—C1—H1	123.6
C18—C13—C11	121.58 (12)	C2—C3—N1	106.02 (15)
C6—C5—C4	119.68 (13)	C2—C3—H3	127.0
C6—C5—H5	120.2	N1—C3—H3	127.0
C4—C5—H5	120.2	C3—C2—N2	111.20 (14)
C11—C10—C7	132.31 (14)	C3—C2—H2	124.4
C11—C10—H10	113.8	N2—C2—H2	124.4
C7—C10—H10	113.8		
			
C8—C9—C4—C5	0.4 (2)	C10—C7—C8—C9	179.97 (14)
C8—C9—C4—N1	−178.68 (13)	C15—C16—N4—O1	0.2 (2)
C1—N1—C4—C9	38.6 (2)	C17—C16—N4—O1	179.89 (15)
C3—N1—C4—C9	−144.54 (16)	C15—C16—N4—O2	−179.52 (15)
C1—N1—C4—C5	−140.54 (16)	C17—C16—N4—O2	0.2 (2)
C3—N1—C4—C5	36.3 (2)	C14—C13—C18—C17	−1.2 (2)
C15—C14—C13—C18	1.4 (2)	C11—C13—C18—C17	179.44 (14)
C15—C14—C13—C11	−179.21 (14)	C17—C16—C15—C14	−0.1 (2)
C10—C11—C13—C14	170.92 (14)	N4—C16—C15—C14	179.55 (14)
C12—C11—C13—C14	−9.6 (2)	C13—C14—C15—C16	−0.8 (2)
C10—C11—C13—C18	−9.7 (2)	C15—C16—C17—C18	0.4 (2)
C12—C11—C13—C18	169.74 (14)	N4—C16—C17—C18	−179.33 (14)
C9—C4—C5—C6	−0.2 (2)	C13—C18—C17—C16	0.3 (2)
N1—C4—C5—C6	178.97 (14)	C10—C11—C12—N3	−178 (2)
C12—C11—C10—C7	−0.7 (3)	C13—C11—C12—N3	3 (2)
C13—C11—C10—C7	178.73 (14)	C2—N2—C1—N1	0.59 (19)
C6—C7—C10—C11	−174.72 (15)	C3—N1—C1—N2	−0.18 (19)
C8—C7—C10—C11	5.5 (3)	C4—N1—C1—N2	177.24 (14)
C4—C5—C6—C7	−0.1 (2)	C1—N1—C3—C2	−0.32 (18)
C8—C7—C6—C5	0.1 (2)	C4—N1—C3—C2	−177.72 (14)
C10—C7—C6—C5	−179.70 (14)	N1—C3—C2—N2	0.7 (2)
C4—C9—C8—C7	−0.5 (2)	C1—N2—C2—C3	−0.8 (2)
C6—C7—C8—C9	0.2 (2)		

### Hydrogen-bond geometry (Å, º)

**Table:**

*D*—H···*A*	*D*—H	H···*A*	*D*···*A*	*D*—H···*A*
C18—H18···O2^i^	0.93	2.54	3.464 (2)	173

Symmetry code: (i) *x*, −*y*+3/2, *z*+1/2.
