# Supplementary figures and images for: Crystal structure of 3-[4-(1H-imidazol-1-yl)phen­yl]-2-(4-nitro­phen­yl)prop-2-ene­nitrile
Source: Acta Crystallogr E Crystallogr Commun. 2015 Aug 6;71(Pt 9):o635. doi: 10.1107/S2056989015013730 (PMC4555437; doi:10.1107/S2056989015013730)

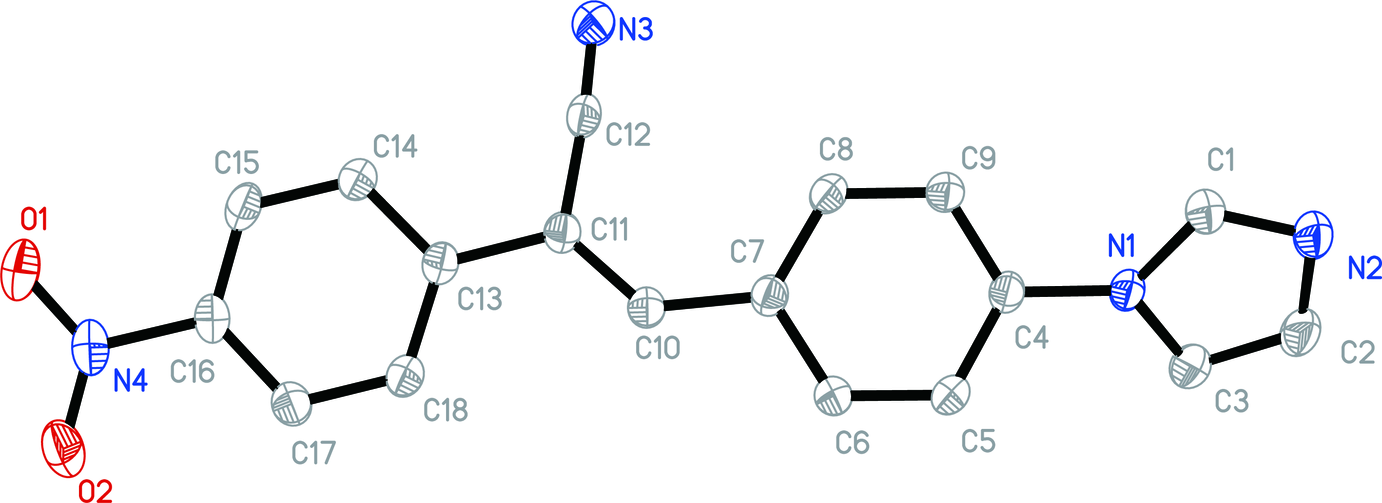

Supplement: Supplementary file 4 [file e-71-0o635-fig1.tif]

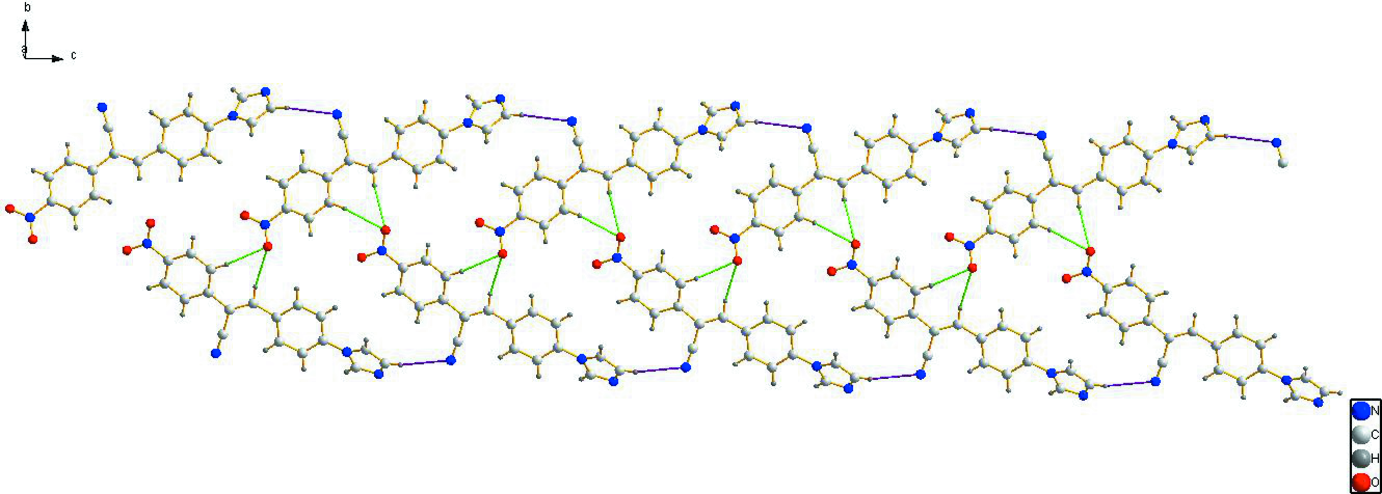

Supplement: Supplementary file 5 [file e-71-0o635-fig2.tif]
